# Effect of heat treatment on microstructure and mechanical properties of selective laser melted Inconel 718 alloy

**DOI:** 10.1371/journal.pone.0309156

**Published:** 2024-09-05

**Authors:** Qiuxia Fan, Jianyu Li, Liuwei Zheng, Caiyun Hao, Qianqian Zhang, Yingzhi Wang

**Affiliations:** 1 School of Automation and Software Engineering, Shanxi University, Taiyuan, China; 2 College of Materials Science and Engineering, Taiyuan University of Technology, Taiyuan, China; 3 Instrumental Analysis Center, Taiyuan University of Technology, Taiyuan, China; Universidade Federal do Rio Grande do Sul, BRAZIL

## Abstract

In this study, we conducted two heat treatment processes, namely double aging (DA) and solid solution followed by double aging (SA), on the Inconel 718 alloy fabricated by selective laser melting (SLM). The aim was to investigate the microstructure evolution and mechanical properties of Inconel 718 under different heat treatment conditions. To achieve this, we employed advanced techniques such as Scanning Electron Microscope (SEM), electron backscattered diffraction (EBSD), energy dispersive spectroscopy (EDS), x-ray diffraction (XRD), Tofwerk time-of-flight secondary ion mass spectrometer (TOF-SIMS), and transmission electron microscopy (TEM). Our experimental findings reveal the presence of cellular high-density dislocation substructures in the as-received (AR) specimens, with a significant accumulation of Laves phase precipitates at grain boundaries and subgrain boundaries. After the DA treatment, the cellular substructure persists, with higher concentrations of γ" and γ’ strengthened phases compared to AR specimen. Conversely, the SA specimen undergoes almost complete recrystallization, resulting in the dissolution of brittle Laves phases and a substantial increase in the content of strengthening phase γ’’ and γ’. As a consequence of the precipitation of the γ’’ and γ’ strengthened phase and the modification of the microstructure, the material exhibits enhanced strength and hardness, albeit at the expense of reduced plasticity. The investigation of the relationship between heat treatment processes and precipitation behavior indicates that the SA heat treatment yields favorable mechanical properties that strike a balance between strength and plasticity.

## 1. Introduction

Inconel 718 is an iron-nickel-based high-temperature alloy strengthened through precipitation [1]. It finds extensive applications in the aviation, aerospace, oil, gas, and nuclear industries due to its exceptional tensile strength, fatigue strength, creep strength, fracture strength, high-temperature oxidation resistance, and stable chemical properties at low temperatures [2–4]. Traditionally, Inconel 718 alloy is manufactured through casting or forging methods [5]. However, as aerospace and other industrial sectors rapidly evolve, component service environments have become increasingly complex, demanding enhanced performance. The conventional manufacturing techniques tend to produce defects such as segregation and flakes, leading to material failure [6]. Moreover, the high cost and long production cycle associated with casting and forging methods make them unsuitable for the manufacturing of complex small-batch parts [7]. Consequently, a novel approach is required to meet production demand and achieve the desired properties of Inconel 718 alloy is needed.

Additive manufacturing (AM) is an advanced molding technology that seamlessly integrates computer-aided design, material processing, and molding techniques. It leverages 3D modeling to fabricate solid parts through the layer-by-layer stacking of materials using extrusion, sintering, melting, and light-curing [8–10]. This remarkable technology has demonstrated success in the production of Inconel 718 alloy, owing to its exceptional flexibility, absence of material size and shape limitations, efficient material utilization, and rapid production cycle time [11–13]. Specifically, Selective laser melting (SLM) stands out as a type of additive manufacturing that employs low laser power, fast scanning speeds, small laser beam diameters, and minimal heat input, which are essential for the process. With SLM, complex parts can be produced directly in a short cycle time [14, 15].

Inconel 718 derives its strengthen from the γ’ (Ni_3_(Al, Ti)) phase and γ’’ (Ni_3_Nb) phase, with the γ’’ phase serving as the primary strengthening phase [16–19]. While SLM technology offers significant advantages in manufacturing parts, the intricate Marangoni convection and extremely rapid cooling rate (10^3^–10^8^ K/s) during the manufacturing process result in the concentration of residual stress, segregation of refractory elements like Nb and Mo and precipitation of Laves brittle phases (A_2_B) and carbides (MC, M_6_C, M_23_C) [20–23]. Due to the existence of these defects, the precipitation of the strengthening γ′ and γ′′ phases is initiated, and the mechanical properties are decreased, which could lead to failure of IN718 products and greatly limit their applications [24]. Therefore, to meet the product performance requirements of the material, appropriate heat treatment is necessary. Heat treatment serves to eliminate brittle phases, facilitate the precipitation of γ’’ and γ’ strengthening phases, homogenize the microstructure, and alleviate residual stress induced by additive manufacturing. Traditional heat treatment processes for cast and forged Inconel 718 alloys often adhere to the American Aerospace Materials Specification (AMS), specifically AMS 5383 and AMS 5662 [25]. Due to the unique microstructure of alloys formed through SLM, the conventional heat treatment process cannot be directly applied to SLM-formed Inconel 718 alloys. Consequently, numerous scholars have made process improvements to improve the overall performance of the alloy material. Multiple researchers have worked on improve the manufacturing processes which influence alloy materials’ overall performance. Wan et al. [26] investigated the impact of heat treatment on the fatigue strength of Inconel 718 alloy. Their findings revealed that appropriate solution treatment can induce the formation of ultrafine-scale acicular phases, effectively mitigating the detrimental effects of voids and improving the fatigue strength of the alloy. Similarly, Huang et al. [27] conducted a study on the microstructure, precipitation phases, and tensile properties of SLM-formed Inconel 718 alloy under various aging temperatures. The results demonstrated that the dissolution of the brittle δ phase and improved precipitation strengthening by the γ" and γ’ phases are the main factors behind of enhanced tensile strength at the increasing aging temperature.

The heat treatment process employed in this paper was designed according to the precipitation temperature and dissolution temperature of the precipitated phase of the material and the time-temperature transformation (TTT) curve drawn by Brooks and Bridges [28, 29]. The samples were treated by direct double aging, solution followed by double aging. To accurately characterize the precipitates in the samples, a comprehensive range of analytical techniques including SEM, EBSD, EDS, XRD, TOF-SIMS, TEM, and others were employed. In this work, in order to elucidate the effects of heat treatment on microstructure and mechanical properties of SLM Inconel 718 alloy, samples in direct double aging and solution followed by double aging states with different microstructure characterization were investigated.

## 2. Experimental materials and methods

### 2.1 Materials and SLM processes

**Fig 1:**
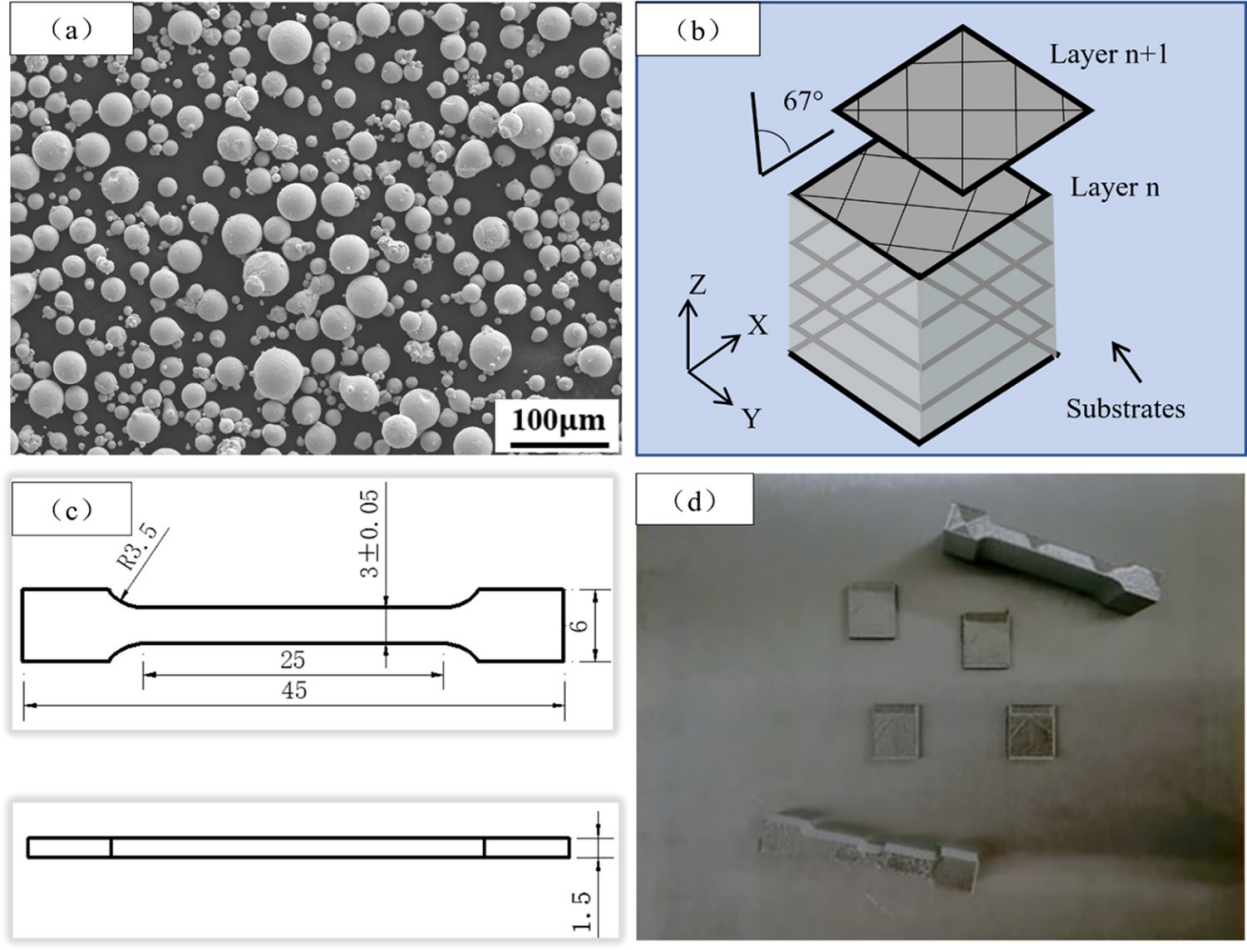
(a) SEM image of processed Inconel 718 powder; (b) Schematic diagram of laser scanning strategy of SLM sample; (c) Stretching specimen dimensions; (d) SLM molding sample.

The material utilized in this experiment was produced using Micron Speed Build FF-M140-H equipment, employing Inconel 718 spherical powder with the chemical composition outlined in Table 1. The SEM image of the powder is displayed in Fig 1(A). Based on extensive experimental data, the SLM forming process parameters were carefully selected as follows: laser power of 200 W, scanning pitch of 0.1 mm, scanning rate of 1000 mm/s, and layer thickness of 30 μm. The checkerboard format scanning strategy was employed, with each successive layer rotated by 67° along the Z-axis, as depicted in Fig 1(B). A stainless-steel substrate was used for the forming process. To minimize stress during printing, the substrate was preheated to 80°C prior to forming. The specimens were subsequently cut from the substrate to the required thickness using an electrical discharge machine (EDM) wire cutting, with a tissue specimen size of 10 mm × 12 mm × 2 mm and a tensile specimen thickness of 1.5 mm, the dimensions of which are shown in Fig 1(C). Fig 1(D) shows a sample formed directly on the substrate by SLM.

**Table 1:** Chemical composition of Inconel 718 alloy powder (wt.%).

Ni	Cr	Mo	Nb	Al	Ti
50.00~55.00	17.00~21.00	2.80~3.30	4.80~5.50	0.02~0.80	0.65~1.15
C	S	Si	Mn	P	Fe
0.02~0.06	≤0.015	≤0.35	≤0.35	≤0.015	Bal

According to relevant references [30], the precipitation and dissolution temperature ranges for the phases in Inconel 718 alloy are as follows: the γ’ phase precipitates between 593~816°C and dissolves between 843~871°C; the γ’’ phase precipitates between 595~870°C and dissolves between 870~930°C; the δ phase precipitates between 780~980°C and dissolves between 980~1020°C; and the Laves phase almost completely dissolves above 1100°C. To investigate the influence of heat treatment on the microstructure and mechanical properties of the alloy, and to explore the relationship between microstructure and properties, heat treatment processes were designed based on the precipitation and dissolution temperature ranges of each phase to control phase precipitation. Therefore, the deposited specimens were heat-treated using an XD-1400ST high-temperature tube furnace, with process parameters shown in Table 2.

**Table 2:** Heat treatment process for Inconel 718 alloy specimens.

Specimens	Heat treatment processes
**As received (AR)**	/
**Double aging (DA)**	720°C for 8h→Temperature reduction at 50°C/h, furnace cooling for 2h→620°C for 8h→Air cooling to room temperature
**Solution followed by double Aging (SA)**	1150°C for 2h, Water cooling→DA

### 2.2 Microstructure characterization

The samples were analyzed using a Tescan S8000 field emission scanning electron microscopy equipped with energy dispersive spectroscopy and electron backscattered diffraction techniques. For SEM sample preparation, electrolytic corrosion was performed using a 10% H_2_C_2_O_4_ solution at a corrosion voltage of 5 V and a corrosion time of 15 s. During the SEM test, the voltage was set to 15 kV and the beam current to 1 nA. For EBSD sample preparation, electropolishing was carried out for 70 s with a 10% HClO_4_ solution at a voltage of 30 V. The EBSD test was conducted at a voltage of 20 kV and a beam current of 3 nA. Subsequently, the samples were analyzed by X-ray diffraction (XRD) using a KY-2000 X-ray diffraction analyzer. The XRD test parameters included a voltage of 40 kV, a current of 40 mA, and a step size of 4°/min. The microstructure and element distribution of the samples were characterized using JEM-F200 ultra-high resolution transmission electron microscopy (TEM) and TOF-SIMS. TEM testing employed electrochemical twin-jet polishing, with the electrolyte being a 10% HClO_4_ solution in alcohol. The electrolysis was conducted at -12.5°C with an electrolytic voltage of 28 V and a photometric value of 150. The TOF-SIMS operated at a working voltage of 30 kV with an ion beam current of 250 pA.

### 2.3 Mechanical property testing

Tensile tests were carried out at room temperature using a universal testing machine, with a displacement rate of 0.5 mm/s, to assess the variations in strength and plasticity of the material under different heat treatment processes. To ensure experiments accuracy, each set of underwent three repetitions of testing. The Vickers hardness of specimens in different heat treatment states was measured using a Vickers hardness tester with a load of 500 gf and a loading time of 15 s. Each group of specimens was tested 10 times and the average value was taken.

## 3. Results and discussions

### 3.1 Microstructure evolution

**Fig 2:**
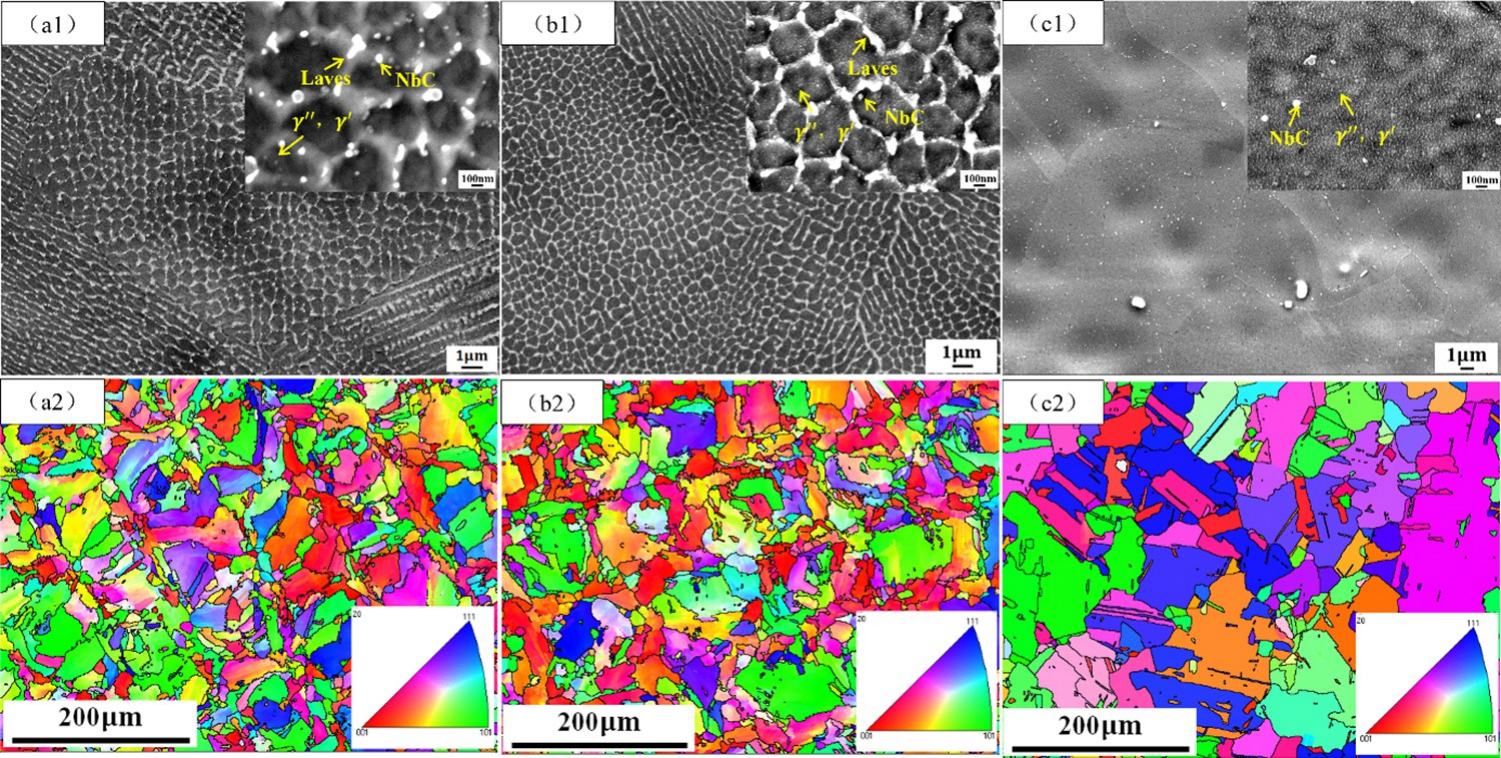
Microstructures of the AR(a1,a2), DA(b1,b2), and SA(c1,c2) in the X-Y direction: SEM images of the AR(a1), DA (b1), and SA(c1) samples; EBSD-IPF maps of the AR(a2), DA (b2), and SA(c2) samples.

Fig 2 shows present SEM and EBSD micrographs of the of AR, DA, and SA samples in X-Y direction. In Fig 2(a1), the SEM images of specimen AR reveal a cellular substructure with a diameter of about 500 nm, deviating from the conventional organization observed in cast and wrought alloys. Notably, grain boundaries and subgrain boundaries exhibit abundant white irregular mass and irregular spherical precipitation phases, ranging in diameters of 50 to 100 nm. Additionally, near the subgrain boundaries, a few spherical precipitation phases with diameters of 10 to 20 nm are also observed. Fig 2(a2) illustrates the IPF diagram of sample AR. It is observed that there are about 100μm wide and 67° crisscross distribution of melting channels in the diagram. During the process of laser scanning metal powder, there is a lap remelting zone between the adjacent laser, which is prone to recrystallization and produces fine grains. The IPF diagram further demonstrates that the grains in specimen AR are approximately equiaxed, exhibiting an alternating pattern of thickness and fineness. These microstructure characteristics are consistent with the scanning strategy. This is consistent with previous research by Li et al [5]. Fig 2(b1) presents the SEM diagram of the DA sample. In comparison to Fig 2(a1), irregular massive and circular particles still exist within the cellular substructure grain boundaries of the DA specimen. However, a notable difference is the presence of numerous spherical phases with diameters ranging from approximately 10 to 20 nm precipitated within the cellular substructure. Fig 2(b2) shows the IPF diagram of sample DA in the X-Y direction. Compared with Fig 2(a2), it can be seen that the grain growth direction and size of sample DA after heat treatment have no significant change, which is similar to sample AR. Fig 2(c1) is the SEM diagram of sample SA. Compared with samples AR and DA, the cellular substructures of sample SA disappeared completely after solution treatment at 1150°C. The grain size is distinct, exhibiting a close-to-equiaxed shape. There are a few irregular round white precipitates with a diameter of about 50nm and a large number of dispersed white spherical precipitates with a diameter of about 10 ~ 20 nm. Fig 2(c2) shows the IPF diagram of sample SA. By comparison, with Fig 2(a2) and 2(b2), crisscross fusion channels in the sample completely disappear, the grains grow significantly, the shape is close to equiaxed, and twin crystals appear.

Using the Channel 5 software for EBSD data analysis, the crystallite sizes of samples under different heat treatment conditions were statistically analyzed. The results showed that in the X-Y direction, the average crystallite size of sample AR was 11.407 μm, sample DA was 11.316 μm, and sample SA was 13.651 μm. It is evident from the comparison that compared to sample AR, sample DA exhibited no significant change in crystallite size, while sample SA showed a noticeable increase in crystallite size.

**Fig 3:**
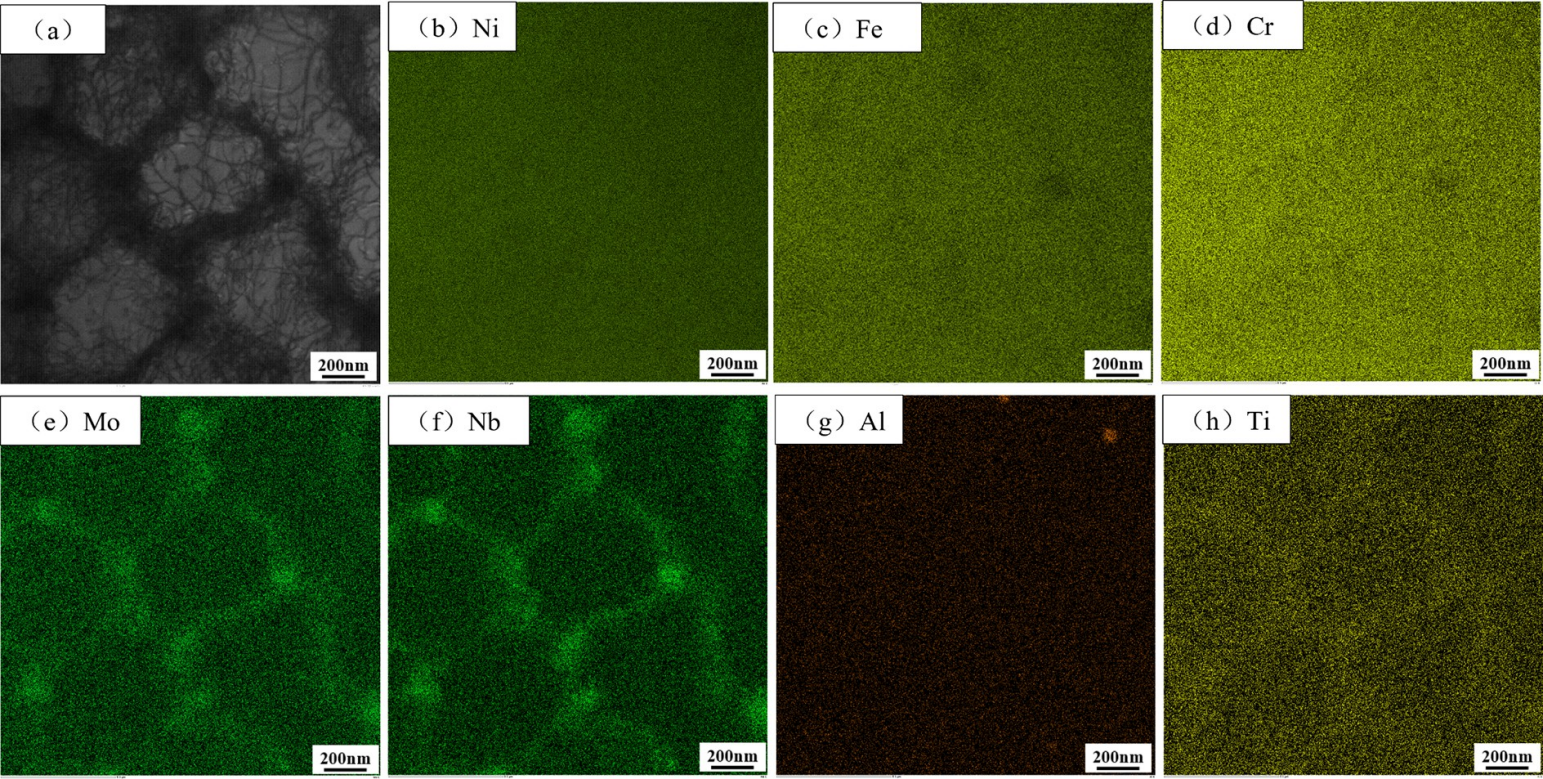
EDS element distribution maps of the cellular substructure of sample AR in the X-Y direction.

Fig 3 shows the EDS element distribution of samples captured by transmission electron microscopy in STEM mode. In Fig 3(A), black massive precipitates were observed at the subgrain boundaries of cellular structures and a large number of dislocation entanglements existed. It can be observed from Fig 3(E), 3(F) and 3(H) that segregation of Mo, Nb, and Ti elements exists at the subgrain boundaries of the specimen, especially at some triangular grain boundaries where the segregation is most severe. According to EDS analysis and related literature [31–33], these irregular masses with a width of about 100 nm precipitated out as Laves phase and irregular spheres with a diameter of about 50 nm precipitated out as NbC particles.

**Fig 4:**
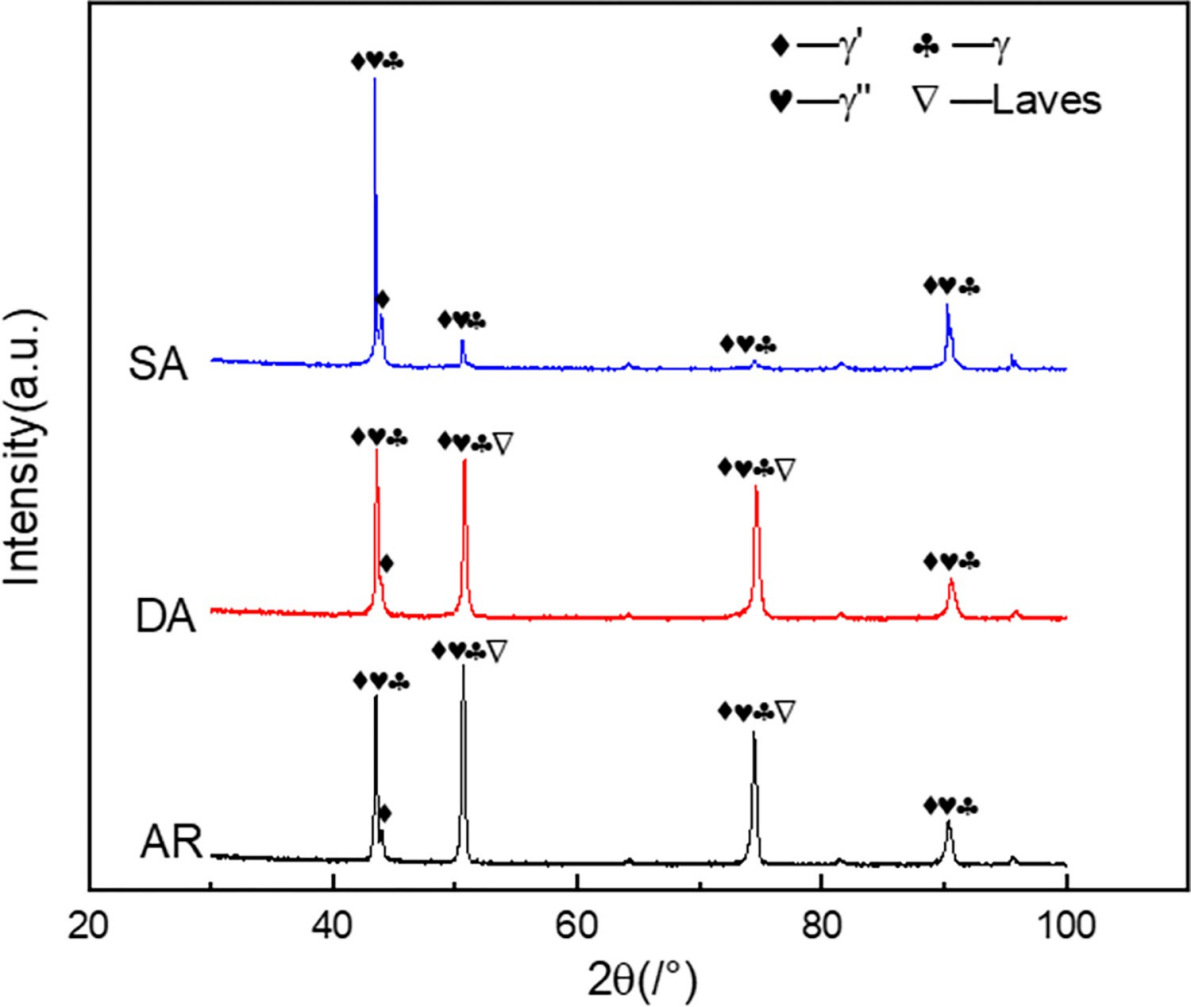
XRD patterns of specimens in different heat treatment states.

To determine the type and content of the precipitated phase in the samples under different heat treatment conditions, XRD phase analysis was conducted on the samples. The XRD pattern of the sample is displayed in Fig 4. The data were analyzed and processed using Jade software to identify the phase corresponding to each prominent peak. From XRD data of sample AR, it is evident that, in addition to matrix γ phase, Laves phase, γ" and/or γ’ phases are present. γ" and γ’ phases cannot be distinguished by XRD. Compared with sample AR, the peak intensity corresponding to Laves phase in sample DA decreased, and the corresponding peak intensity corresponding to γ" and/or γ′ increased. It can be observed from the XRD pattern of sample SA that the Laves phase has disappeared, and the peak intensity corresponding to γ" and/or γ′ is significantly increased. The observed variation of peak value corresponding to Laves is consistent with that observed in SEM. The white spherical precipitates with a diameter of about 10 ~ 20 nm in the sample can also be preliminarily determined as γ" and/or γ’ by XRD pattern analysis.

**Fig 5:**
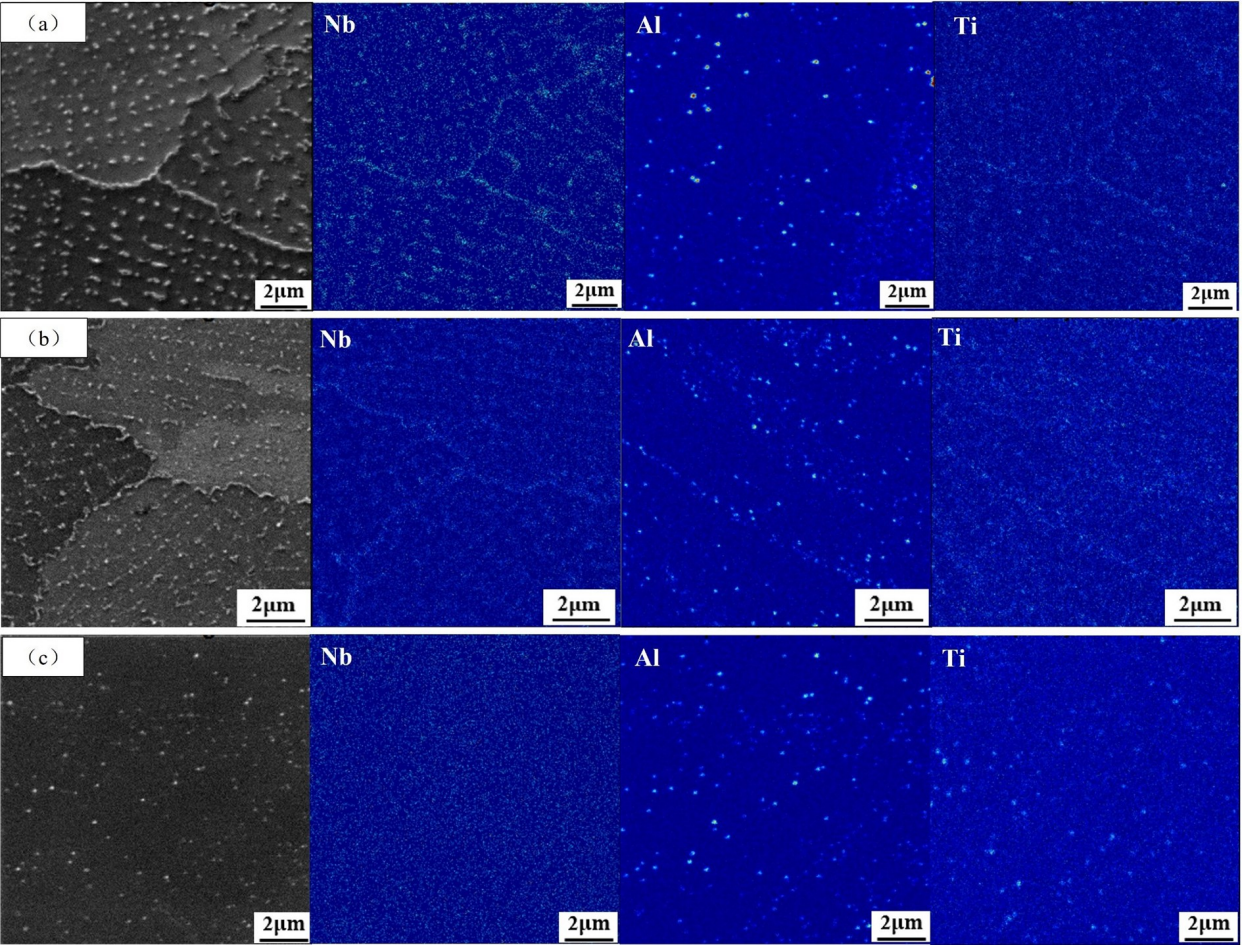
Element distribution map: (a) AR; (b) DA; (c) SA.

TOF-SIMS was employed to conduct a comprehensive analyze the distribution of elements in the sample. Fig 5 presents the obtained distribution diagram of sample elements obtained using TOF-SIMS. Fig 5(A) shows the element distribution of sample AR. Segregation of Nb and Ti elements was observed at grain boundaries and subgrain boundaries. The segregation of the Al element shows a point distribution. From the segregation of Nb, Al, and Ti elements, we can judge the existence of the spherical γ’ phase here. Moving on to Fig 5(B), it displays the element distribution of sample DA. It is noticeable that the distribution of element Nb is similar to that of sample AR, with enriched at grain boundaries and subgrain boundaries. The segregation of Al and Ti elements in the sample appears as discrete points. Fig 5(C) portrays the element distribution of sample SA. As the size of the precipitated phase is small and the content of the Nb element in the matrix is relatively large, no obvious segregation phenomenon of the Nb element can be seen in the picture. However, the distribution of Al and Ti elements in the samples is similar to that of DA samples, both of which are point segregation. By comparing Fig 5(A)–5(C), it is observed that Al and Ti segregation increase in the samples after heat treatment, and the number of SA segregation is more than that of DA.

**Fig 6:**
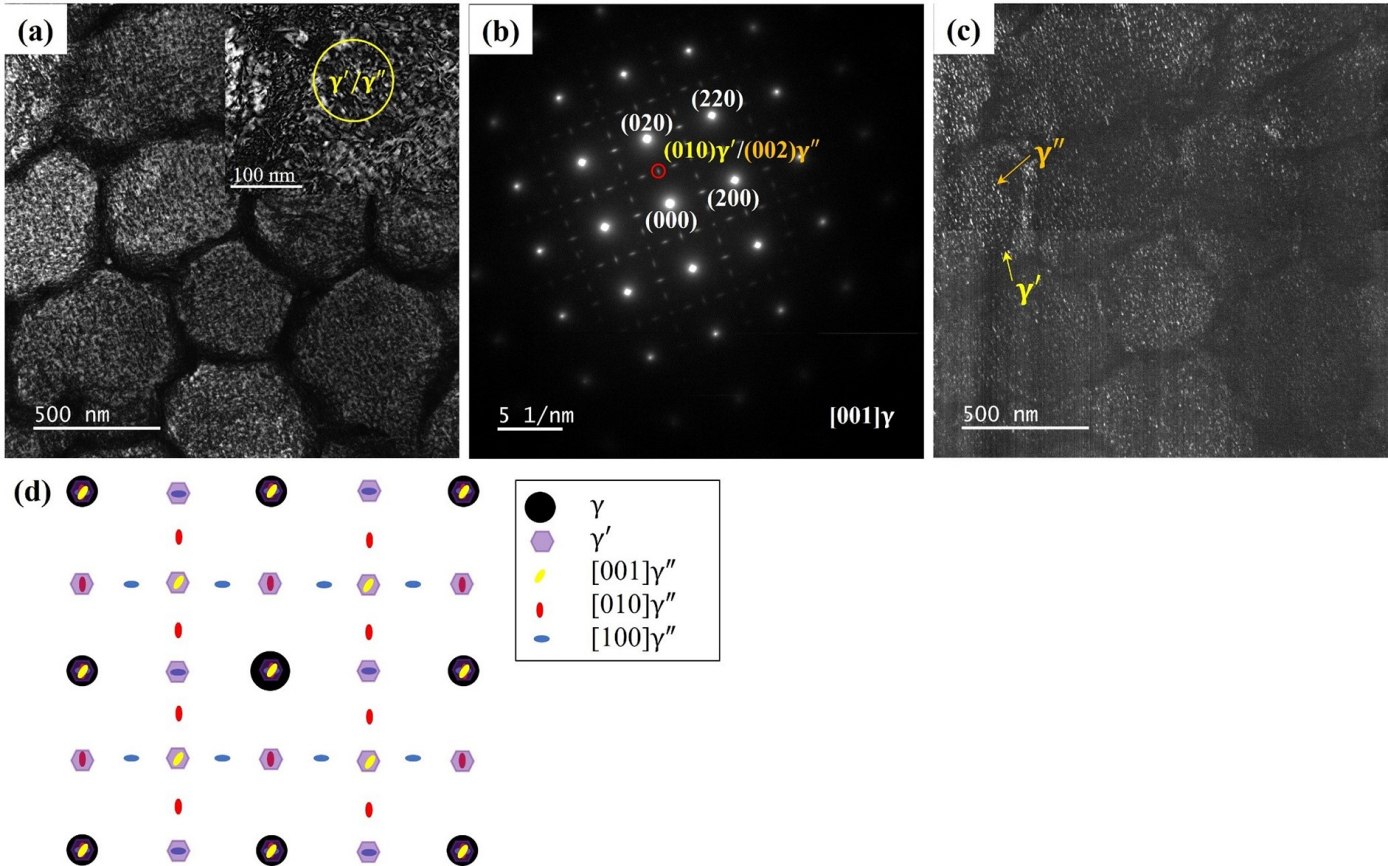
TEM test of DA sample: (a) TEM bright-field image; (b) SAED patterns along [1] γ zone axis; (c) TEM dark-field image; (d) SAED diagram.

The aforementioned testing methods did not provide a precise visual characterization of the precipitated phase visually, necessitating the use TEM for further distinguish. Fig 6(A) is a bright-field (BF) TEM image showing cellular sub-structures with high-density dislocations in the DA specimen. Selected area electron diffraction (SAED) is carried out on the enlarged area in the illustration in Fig 6(A). The corresponding SAED pattern along the [1] γ zone axis is shown in Fig 6(B), it is found that there are γ" and γ’ phase diffraction spots in the γ matrix. Fig 6(C) exhibits a dark-field (DF) image corresponding to the diffraction spot in the red circle in Fig 6(B). The figure clearly illustrates the presence of the spherical γ’ phase and disk γ" phase. Previous scholars have pointed to overlapping SAED patterns in certain directions [2, 5, 34]. By combining these with previous studies, SAED can be accurately calibrated, as shown in Fig 6(D).

### 3.2 Mechanical properties

**Fig 7:**
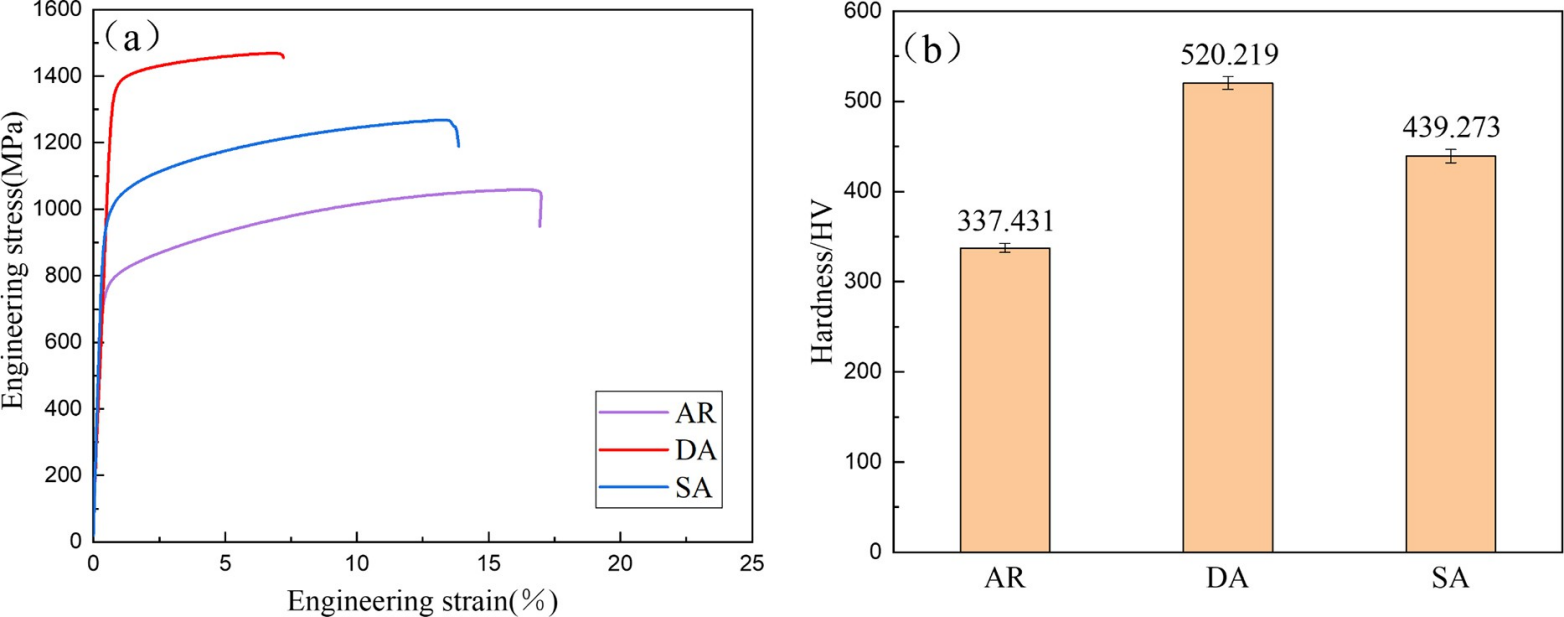
(a) Stress-strain curves; (b) Vickers hardness.

Fig 7(A) presents the stress-strain curves of Inconel 718 alloy specimens subjected to different heat treatment processes, while Table 3 provides the corresponding mechanical properties. It can be seen that the specimen directly enters the plastic deformation stage until fracture after the elastic deformation stage, and there is no obvious yield stage. Compared with specimen AR, the strength of specimens DA and SA increased and the plasticity decreased. Specifically, the specimen DA tensile strength increased by 38.7%, yield strength increased by 79.1% and elongation decreased by 57.4%. The specimen SA tensile strength increased by 19.8%, yield strength increased by 26.8% and elongation decreased by 17.8%.

**Table 3:** Mechanical properties of alloy specimens under different heat treatment states.

Specimens	Tensile strength (MPa)	Yield strength (MPa, 0.2%)	Elongation (%)
**AR**	1060±26	765±38	16.9±1.3
**DA**	1470±30	1370±10	7.2±1.5
**SA**	1270±32	970±5	13.9±2.1

Fig 7(B) shows the Vickers hardness of Inconel 718 alloy specimens subjected to various heat treatment conditions. The image reveals that the AR specimen exhibits a hardness of 337.431 HV, and after undergoing different heat treatment processes, the hardness of the specimen increases to varying degrees. The hardness of DA is 520.219 HV, which is 54.2% higher than that of AR. The hardness of SA is 439.273 HV, which is 30.2% higher than that of AR.

## 4. Discussion

### 4.1 Formation of the microstructure of Inconel 718 alloy formed by SLM

**Fig 8:**
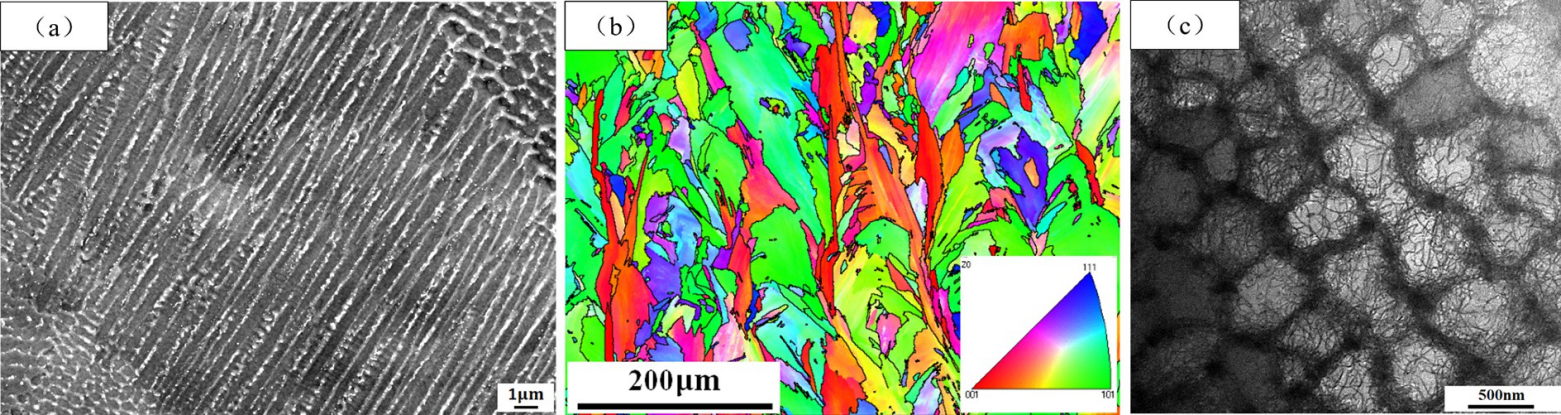
(a) SEM image of sample AR in X-Z direction; (b) IPF map of sample AR in X-Z direction; (c) Cellular substructures are seen in the TEM.

To analyze the cause of cellular substructure in SLM molding materials, the X-Z direction microstructure of AR samples was characterized. Fig 8(A) displays an SEM image of AR sample in the X-Z direction, revealing the presence of numerous columnar subgrains with a width of approximately 500 nm. Fig 8(B) shows the IPF map of specimen AR in the X-Z direction. The grain grows columnar, and its growth direction is parallel to the X-Z direction. Compared with Fig 2(a1), it can be observed that the grain shape of Inconel 718 alloy samples formed by SLM is irregular, and there are a large number of subgrains in the grains. Notably, distinct microstructural variations are observed between the X-Y and X-Z directions. In the X-Y direction, a large number of cellular subgrains were observed within the grains, and a small number of columnar subgrains existed in the remelted zone (Fig 2a1). However, the grain structure in the X-Z direction is reversed. The subgrains inside the grains are mostly columnar, and a few cellular subgrains exist in the remelting zone (Fig 8(A)).

The formation of the unique microstructure in SLM is primarily due to the high temperature gradient caused by rapid heating and cooling during the molding process [35]. During the laser scanning process, a significant temperature gradient is generated in the X-Z direction of the molded part, leading to rapid grain growth along the temperature gradient and the formation of columnar grains. However, the temperature gradient in the remelting overlap zones of SLM alloys is more chaotic, resulting in the growth of columnar grains in multiple directions. Consequently, when observed from the X-Y direction, the cellular and other shapes seen are the cross-sections of columnar grains growing in different directions. This is also indicated by the fact that the average width of columnar subgrains and the average diameter of cellular subgrains are almost the same. The formation of this special substructure can improve the deformation resistance of the alloy.

Fig 8(C) exhibits the observed cellular substructure through TEM analysis. It can be seen in the picture that there are a large number of dislocation lines at the matrix and subgrain boundary, and there is a dislocation entanglement at the subgrain boundary, which is larger than the dislocation density in the matrix area. Furthermore, some black irregular shapes precipitate at subgrain boundaries. The reason for the formation of this microstructure is that the solidification speed of the specimen is too fast in the process of molding, which causes a large thermal stress inside the specimen, and then produces a large number of dislocations. The rapid cooling rate hinders the diffusion of chemical components within the metal, leading to element segregation in the alloy. As the element segregation reaches a critical concentration, precipitation occurs. This precipitation further immobilizes the dislocations, thereby increasing the dislocation density at the subgrain boundaries. According to the analysis in Part 3, the irregular precipitates precipitated at the subgrain boundaries are Laves phase.

### 4.2 Effect of different heat treatments on the precipitated phase

The Inconel 718 alloy primarily consists of several phases, including the γ phase, strengthening γ’ phase, γ’’ phase, brittle δ phase, Laves phase, and carbide. The solidification process of Inconel 718 alloy formed by SLM is as follows: L→L+*γ*→L+*γ*+NbC→*γ*+NbC+Laves [36]. The precipitation of the alloy material takes a certain time, while the solidification rate of the alloy formed by SLM is fast, and precipitation is limited. As a result, the main phases in the sample AR are the matrix γ phase and brittle Laves phase, as shown in Fig 2(a1). Secondly, a small amount of γ" and γ′ strengthened phase precipitated during the cooling process. In order to control the microstructure of the alloy and optimize its performance, different heat treatment processes were carried out.

Double aging treatment can precipitate more strengthening phases in the alloy. This is consistent with the findings of Lee et al. [3]. The specimens were held at 720°C and 620°C for 8 hours each, allowing sufficient time for the precipitation of strengthening phases γ" and γ’. A comparison of SEM images of specimens AR and DA in Fig 2(a1) and 2(b1) shows a significant increase in the number of spherical precipitates in DA. Due to the lower aging temperature, the dissolution temperature of the Laves phase was not reached, hence the Laves phase remains present. To eliminate the influence of brittle phases and further improve the mechanical properties of the alloy, specimen SA underwent solid solution treatment at 1150°C before double aging. The Laves phase in the material completely dissolves and releases a large amount of Nb element. The increase in Nb element promotes the precipitation of Nb-rich strengthening phases γ" and γ’. From Fig 2(c1), it can be observed that in specimen SA, the Laves phase has completely dissolved and disappeared. The specimen contains abundant γ" and γ’ phases along with a small amount of NbC. Since the heat treatment temperature did not stay in the range of δ phase precipitation, no δ phase precipitation was observed during the heat treatment process.

### 4.3 Effect of microstructure and precipitated phase on mechanical properties

Strength and plasticity are crucial indicators for evaluating material properties. Fig 7(A) displays the stress-strain curves of the specimens in different heat treatment states. It can be seen that the tensile strength of sample AR is 1060 MPa, the yield strength is 765 MPa, and the elongation is 16.9%. Following heat treatment, the strength of the specimen DA increased due to the strengthened phase. The tensile strength reached 1470 MPa, an increase of 38.7% compared with the specimen AR, and the yield strength reached 1370 MPa, an increase of 79.1% compared with the specimen AR. However, the precipitation phase acts as pinning points for dislocation motion. After heat treatment, an increase in precipitates at the grain boundaries impedes dislocation motion, leading to dislocation pile-up when dislocations encounter the precipitates. This results in localized stress concentration, making the material prone to fracture. Hence, the plasticity of the material decreases. It can also be seen from the stress-strain curve that the DA elongation of the specimen is 7.2%, which is 57.4% lower than that of the specimen AR.

**Fig 9:**
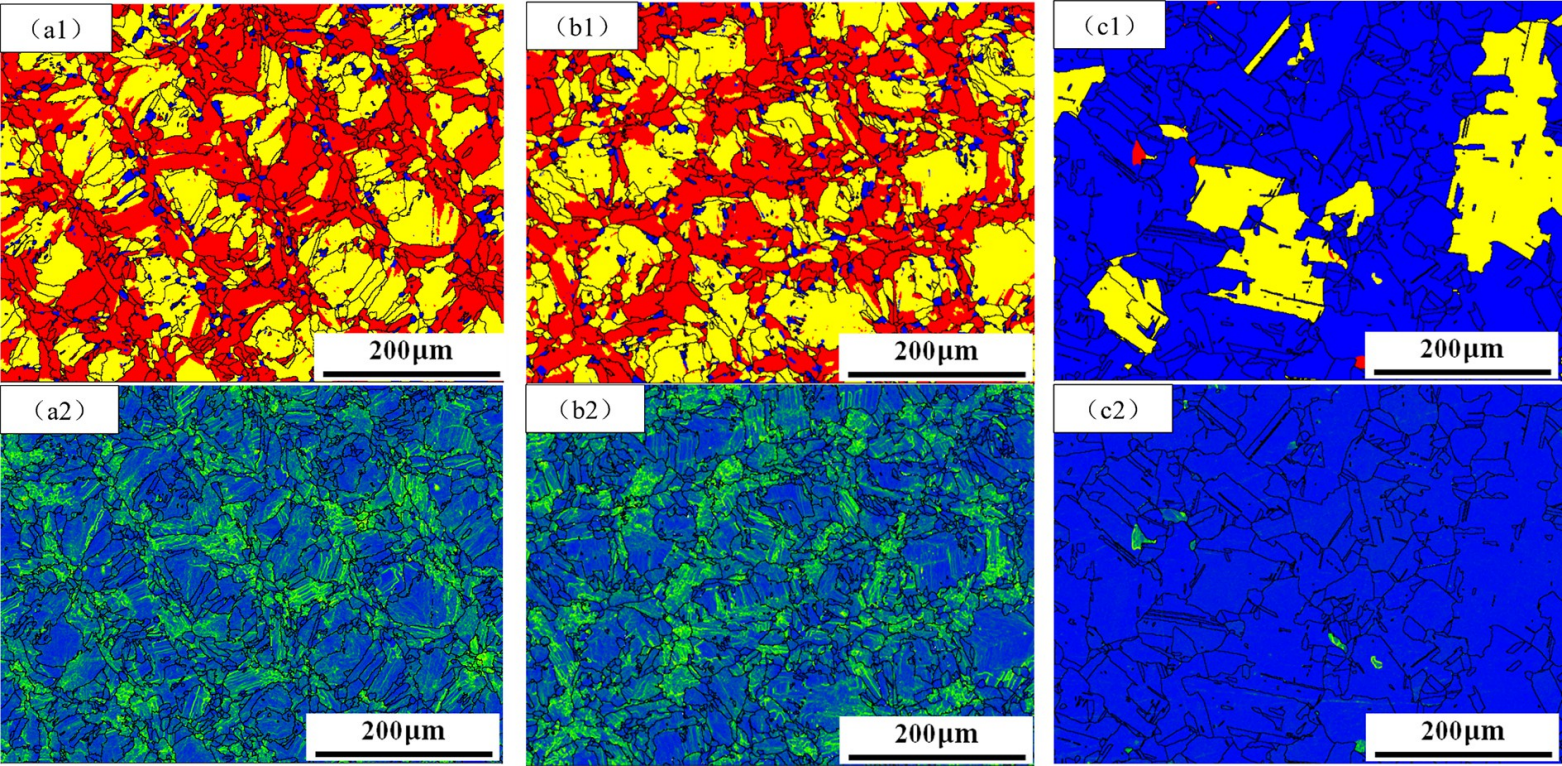
Recrystallization images of the AR(a1), DA (b1), and SA(c1) samples (Blue represents recrystallization, yellow represents substructure, and red represents deformed grains); KAM diagram of the AR(a2), DA(b2), and SA(c2) samples.

In comparison to specimen DA, specimen SA underwent a treatment at 1150°C prior to the double aging heat treatment. This treatment ensured the complete dissolution of the Laves phase in the material, releasing a significant amount of Nb element. Consequently, during the subsequent aging process, a greater quantity of γ" and γ’ strengthened phases precipitated. As a result, the tensile strength of specimen SA reaches 1270 MPa, 19.8% higher than that of specimen AR, and the yield strength reaches 970 MPa, 26.8% higher than that of specimen AR. Fig 9(a1), 9(b1) and 9(c1) is the recrystallization diagram of the specimens under different heat treatment states. By comparing Fig 9(a1) with 9(b1), it can be seen that the recrystallization degree of AR and DA of the specimens is the same, and only a small amount of recrystallization grains exist in the remelting lap area. Fig 9(c1) shows the recrystallization diagram of specimen SA. The specimens are completely recrystallized and the high-density dislocation substructure is completely lost, which leads to a lower strength of SA specimens than DA specimens. Furthermore, the strength and plasticity of the material will be slightly reduced by the obvious growth in grain size of the SA sample carried on by recrystallization. This is because the stress concentration increased as a result of the weakening of the grain boundary’s ability to resist dislocation motion, which promotes deformation inhomogeneity. Moreover, a large amount of γ" and γ’ relative dislocation caused by precipitation from the specimen has a dislocation locking effect, which affects the plasticity of the material [37]. The elongation of the final SA specimen was 13.9%, which was 17.8% lower than the elongation of the specimen AR. High-temperature solid solution treatment can completely dissolve the brittle Laves phase. The elimination of the brittle phase reduces the material’s brittleness and increases its ductility, consequently improving its plasticity. Therefore, although the elongation of specimen SA is lower than that of specimen AR, it is still exceeds that of specimen DA.

From Fig 7(B), the AR hardness of the specimen is 337.431 HV. Following heat treatment using different processes, the hardness of the specimen increases to varying degrees. The hardness of specimen DA was 520.219 HV, which is 54.2% higher than AR. The hardness of specimen SA is 439.273 HV, which is 30.2% higher than that of AR. The increase in hardness is mainly related to the change of precipitated phase content after heat treatment. After heat treatment, the enhanced phase precipitates, which can improve the hardness of materials to a certain extent. The amount of strengthening phase in specimen SA is the largest, and the precipitation strengthening effect should be more significant. However, after the high-temperature solution treatment, the cylindrical and cellular substructures of the specimen SA disappear, reducing the stress and strain of the material, which will hurt the hardness of the material. Fig 9(a2), 9(b2) and 9(c2) shows the kernel average misorientation (KAM) diagram of specimens with different heat treatment states. By comparing Fig 9(a2) and 9(b2), it can be seen that the stress of specimen DA is mainly concentrated in the remelting area, which is slightly lower than that of specimen AR. Fig 9(c2) shows that the stress concentration phenomenon in specimen SA has been eliminated. Moreover, the increase in grain size of the specimen after solution treatment will also affect the hardness of specimen SA. The combined action of these factors makes the hardness increase of specimen SA lower than that of specimen DA.

## 5. Conclusions

(1) SLM molding of Inconel 718 alloy produces irregular grain shapes with high-density dislocation substructures. The microstructure differs between the X-Y and X-Z directions, exhibiting melting channels and columnar and cellular substructures. Double aging treatment fails to eliminate the substructures in sample DA, while high-temperature solution treatment followed by double aging results in near-complete recrystallization in sample SA. This treatment eliminates the melting channels and dislocation cells, increases grain size to an equiaxed morphology, and aligns the microstructure in both directions.

(2) The dominant phases in the deposited Inconel 718 specimen AR are the matrix γ phase and the brittle Laves phase. Direct double aging treatment increases γ" and γ’ phases in DA, while the Laves phase remains mostly unchanged. However, high-temperature solution followed by double aging eliminates the Laves phase completely and significantly enhances γ" and γ’ phases.

(3) The SLM molding method introduces substructures and stress concentrations in the sample, which can be mitigated through heat treatment. This treatment induces the precipitation of strengthened phases, significantly enhancing the strength of Inconel 718 alloy. By adjusting the heat treatment process, the material’s microstructure and phase precipitation can be controlled, optimizing its properties. The solution followed by double aging treatment achieves a tensile strength of 1270 MPa, yield strength of 970 MPa, and plasticity of 13.9%. This process enables a favorable balance between strength and plasticity in the material.

## References

[1] SONG C, MA G, HE A, et al. Multi-phases transformation mechanism of Ti6Al4V/Inconel 718 composite by laser additive manufacturing [J]. Materials Characterization, 2021, 179: 111363.

[2] WANG Y C, LEI L M, SHI L, et al. Effect of heat treatment on strain hardening ability of selective laser melted precipitation-hardened GH4169 superalloy [J]. Materials Characterization, 2022, 190: 112064.

[3] LEE D H, ZHAO Y, LEE S Y, et al. Hydrogen-assisted failure in Inconel 718 fabricated by laser powder bed fusion: The role of solidification substructure in the embrittlement [J]. Scripta Materialia, 2022, 207: 114308.

[4] XU J, HAO Z, FU Z, et al. Hydrogen embrittlement behavior of selective laser-melted Inconel 718 alloy [J]. Journal of Materials Research and Technology, 2023, 23: 359–69.

[5] LI X, SHI J J, WANG C H, et al. Effect of heat treatment on microstructure evolution of Inconel 718 alloy fabricated by selective laser melting [J]. Journal of Alloys and Compounds, 2018, 764: 639–649.

[6] GAYTAN S M, MURR L E, MEDINA F, et al. Advanced metal powder based manufacturing of complex components by electron beam melting [J]. Materials Technology, 2013, 24(3): 180–190.

[7] WANG Z, GUAN K, GAO M, et al. The microstructure and mechanical properties of deposited-IN718 by selective laser melting [J]. Journal of Alloys and Compounds, 2012, 513: 518–523.

[8] PRASAD K, OBANA M, ITO A, et al. Synchrotron diffraction characterization of dislocation density in additively manufactured IN 718 superalloy [J]. Materials Characterization, 2021, 179: 111379.

[9] MOEINFAR K, KHODABAKHSHI F, KASHANI-BOZORG S F, et al. A review on metallurgical aspects of laser additive manufacturing (LAM): Stainless steels, nickel superalloys, and titanium alloys [J]. Journal of Materials Research and Technology, 2022, 16: 1029–1068.

[10] WEI Q, XIE Y, TENG Q, et al. Crack Types, Mechanisms, and Suppression Methods during High-energy Beam Additive Manufacturing of Nickel-based Superalloys: A Review [J]. Chinese Journal of Mechanical Engineering: Additive Manufacturing Frontiers, 2022, 1(4): 100055

[11] SHAHWAZ M, NATH P, SEN I. A critical review on the microstructure and mechanical properties correlation of additively manufactured nickel-based superalloys [J]. Journal of Alloys and Compounds, 2022, 907: 164530.

[12] SCHNEIDER J, LUND B, FULLEN M. Effect of heat treatment variations on the mechanical properties of Inconel 718 selective laser melted specimens [J]. Additive Manufacturing, 2018, 21: 248–254.

[13] MOUSSAOUI K, RUBIO W, MOUSSEIGNE M, et al. Effects of Selective Laser Melting additive manufacturing parameters of Inconel 718 on porosity, microstructure and mechanical properties [J]. Materials Science and Engineering: A, 2018, 735: 182–190.

[14] YANG J, LI F, PAN A, et al. Microstructure and grain growth direction of SRR99 single-crystal superalloy by selective laser melting [J]. Journal of Alloys and Compounds, 2019, 808: 151740.

[15] HOSSEINI E, POPOVICH V A. A review of mechanical properties of additively manufactured Inconel 718 [J]. Additive Manufacturing, 2019, 30: 100877.

[16] TEIXEIRA Ó, SILVA F J G, ATZENI E. Residual stresses and heat treatments of Inconel 718 parts manufactured via metal laser beam powder bed fusion: an overview [J]. The International Journal of Advanced Manufacturing Technology, 2021, 113(11–12): 3139–3162.

[17] AMIRJAN M, SAKIANI H. Effect of scanning strategy and speed on the microstructure and mechanical properties of selective laser melted IN718 nickel-based superalloy [J]. The International Journal of Advanced Manufacturing Technology, 2019, 103(5–8): 1769–1780.

[18] LUO S, HUANG W, YANG H, et al. Microstructural evolution and corrosion behaviors of Inconel 718 alloy produced by selective laser melting following different heat treatments [J]. Additive Manufacturing, 2019, 30: 100875.

[19] M R, KOPPOJU S, TELASANG G, et al. Effect of solutionizing temperature on the microstructural evolution during double aging of powder bed fusion-additive manufactured IN718 alloy [J]. Materials Characterization, 2021, 172: 110868.

[20] KARABULUT Y, TASCIOGLU E, KAYNAK Y. Heat treatment temperature-induced microstructure, microhardness and wear resistance of Inconel 718 produced by selective laser melting additive manufacturing [J]. Optik, 2021, 227: 163907.

[21] YI J H, KANG J W, WANG T J, et al. Effect of laser energy density on the microstructure, mechanical properties, and deformation of Inconel 718 samples fabricated by selective laser melting [J]. Journal of Alloys and Compounds, 2019, 786: 481–488.

[22] CRUZ V, CHAO Q, BIRBILIS N, et al. Electrochemical studies on the effect of residual stress on the corrosion of 316L manufactured by selective laser melting [J]. Corrosion Science, 2020, 164: 108314.

[23] XIE D, LV F, YANG Y, et al. A Review on Distortion and Residual Stress in Additive Manufacturing [J]. Chinese Journal of Mechanical Engineering: Additive Manufacturing Frontiers, 2022, 1(3): 100039.

[24] Yang P, Su H, Guo Y, et al. Influence of cooling rate during the heat treatment process on the precipitates and ductility behavior of inconel 718 superalloy fabricated by selective laser melting [J]. Materials Science & Engineering A, 2024, 902.

[25] DENG D, PENG R L, BRODIN H, et al. Microstructure and mechanical properties of Inconel 718 produced by selective laser melting: Sample orientation dependence and effects of post heat treatments [J]. Materials Science and Engineering: A, 2018, 713: 294–306.

[26] WAN H Y, ZHOU Z J, LI C P, et al. Enhancing Fatigue Strength of Selective Laser Melting-Fabricated Inconel 718 by Tailoring Heat Treatment Route [J]. Advanced Engineering Materials, 2018, 20(10): 1800307.

[27] HUANG J, HUANG Z, DU H, et al. Effect of Aging Temperature on Microstructure and Tensile Properties of Inconel 718 Fabricated by Selective Laser Melting [J]. Transactions of the Indian Institute of Metals, 2022, 75(6): 1403–1410.

[28] BROOKS J, BRIDGES P J S. Metallurgical stability of Inconel alloy 718 [J]. 1988, 88: 33–42.

[29] THOMPSON R, DOBBS J, MAYO D J W J. The effect of heat treatment on microfissuring in alloy 718 [J]. 1986, 65(11): 299.

[30] LIU L, ZHAI C, LU C, et al. Study of the effect of δ phase on hydrogen embrittlement of Inconel 718 by notch tensile tests [J]. Corrosion Science, 2005, 47(2): 355–367.

[31] CHLEBUS E, GRUBER K, KUŹNICKA B, et al. Effect of heat treatment on the microstructure and mechanical properties of Inconel 718 processed by selective laser melting [J]. Materials Science and Engineering: A, 2015, 639: 647–655.

[32] LIU P, HU J, SUN S, et al. Microstructural evolution and phase transformation of Inconel 718 alloys fabricated by selective laser melting under different heat treatment [J]. Journal of Manufacturing Processes, 2019, 39: 226–232.

[33] HUANG L, CAO Y, LI G, et al. Microstructure characteristics and mechanical behaviour of a selective laser melted Inconel 718 alloy [J]. Journal of Materials Research and Technology, 2020, 9(2): 2440–2454.

[34] CAO G H, SUN T Y, WANG C H, et al. Investigations of γ′, γ″ and δ precipitates in heat-treated Inconel 718 alloy fabricated by selective laser melting [J]. Materials Characterization, 2018, 136: 398–406.

[35] KONG D C, DONG C F, WEI S L, et al. About metastable cellular structure in additively manufactured austenitic stainless steels [J]. Additive Manufacturing, 2021, 38: 101804.

[36] TUCHO W M, CUVILLIER P, SJOLYST-KVERNELAND A, et al. Microstructure and hardness studies of Inconel 718 manufactured by selective laser melting before and after solution heat treatment [J]. Materials Science and Engineering: A, 2017, 689: 220–232.

[37] DASH R, BHATTACHARYYA K, BHATTACHARYYA A S. Fracture associated with static and sliding indentation of multicomponent hard coatings on silicon substrates [J]. Fatigue & Fracture of Engineering Materials & Structures, 2023, 46(4): 1641–1645.

